# Prevalence and Correlates of Suicidal Ideation Among Transgender Persons in Delhi, India; a community based cross-sectional study

**DOI:** 10.1192/j.eurpsy.2026.10217

**Published:** 2026-07-31

**Authors:** P. Haldar, P. Gilhotra, R. Sagar, S. K. Rai, R. Khadgawat, P. Kumar, S. Rajan

**Affiliations:** 1 Community Medicine; 2 Psychiatry; 3 Endocrinology and Metabolism, AIIMS New Delhi; 4 programme monitoring, Delhi State AIDS Control Society; 5 Basic Services, National AIDS Control Organization, New Delhi, India

## Abstract

**Introduction:**

Transgender persons face significant health disparities globally. These challenges lead to increased burden of mental health outcomes and suicidal ideation, driven by stigma, discrimination, familial rejection and gender-related issues. There is limited evidence for prevalence of suicidal ideation in India, specifically North India. Thus we aimed to determine the proportion and factors associated with suicidal ideation in last one year among transgender persons of Delhi, India.

**Objectives:**

To determine the proportion and factors associated with suicidal ideation, suicidal planning and suicidal attempt among transgender persons of Delhi, India.

**Methods:**

A cross-sectional study was conducted at the six targeted intervention sites in Delhi, India. These 6 sites provide general support and medical care services to Transgender persons in Delhi. Participants were self-identified transgender persons ≥18 years of age. Structured interview schedule developed using formative research and literature review was administered to assess suicidal ideation, suicidal planning and suicidal attempt during last one year. Factors associated with suicidal ideation were identified in univariable logistic regression; variables associated at p < 0.20 were included in multivariable logistic regression model.

**Results:**

Among the 204 transgender persons with mean±SD age, 27.7±7.9 years, 78 participants (38.2%, 95%CI: 31.6, 44.8) reported having suicidal thoughts in the last 12 months; 55 (27.0%, 95%CI: 20.9, 33.1) had planned for their suicide; 35 (17.2%, 95%CI: 11.7, 22.6) had made at least one suicide attempt. Suicidal ideation was significantly higher among those, with current main occupation of begging with adjusted odds ratio (AOR) 10.37 [2.50,43.0]); cannabis use during past 3 months (AOR=8.4, 95% CI [1.6, 43.4]); encountering more than one stressful events in past 12 months (AOR=3.9, 95% CI [1.2, 13.2]); with having experience of gender dysphoria (AOR= 3.7, 95% CI [1.5, 9.1]); having diagnosed with generalized anxiety disorder (AOR=12.0, 95% CI [2.1, 67.2]).

**Image 1:**
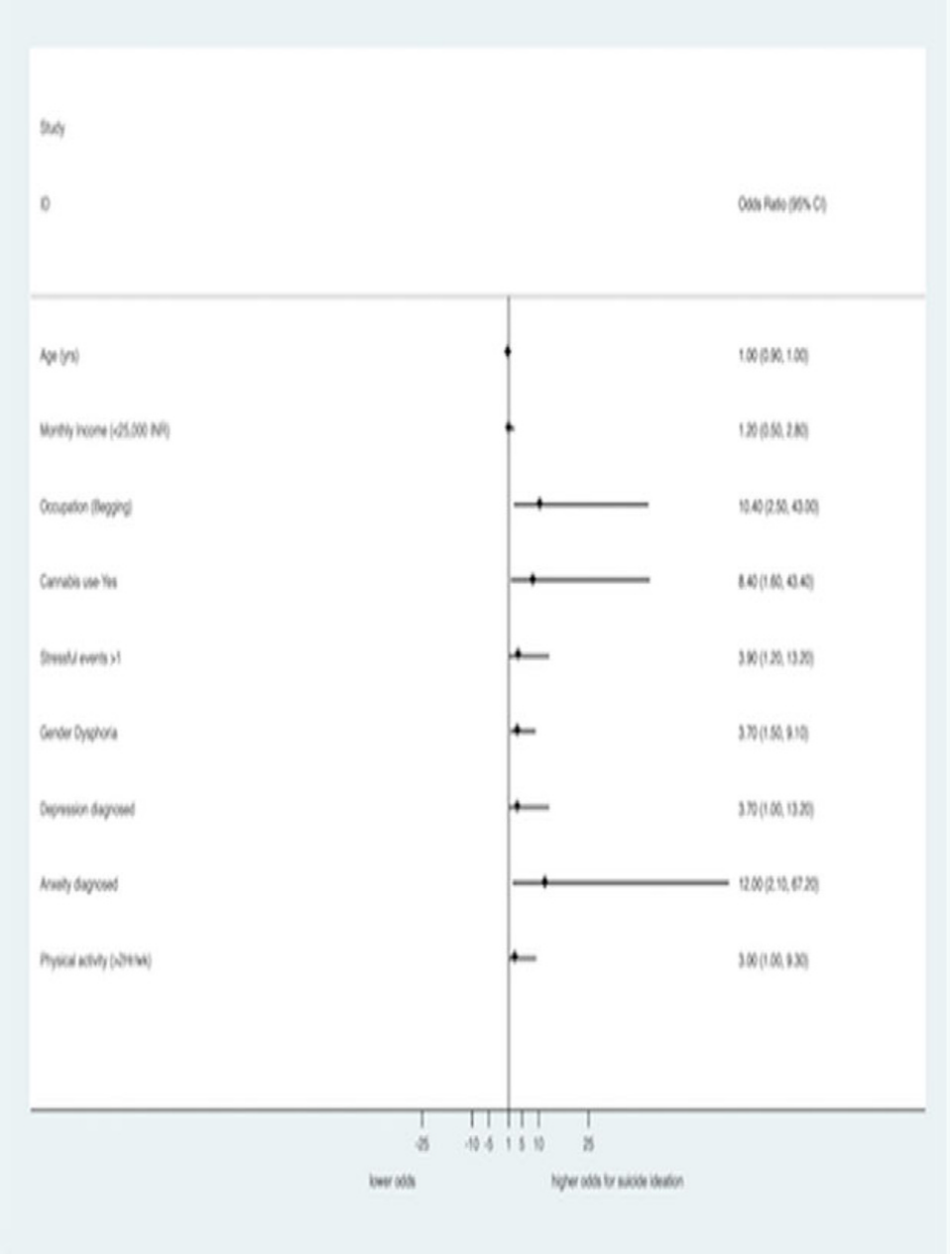
Image 1: Long description.

**Conclusions:**

Suicidal ideation, planning and attempts prevalence rates among transgender persons in Delhi, India are alarming, and significantly asosiated with poor mental health status. Effective mental health and public health policies addressing mental health are urgently needed to prevent suicidal behavior among this highly vulnerable population.

**Disclosure of Interest:**

None Declared

## Long descriptions

### Image 1: Long description

The forest plot consists of a vertical line at the null value of 1.0, with horizontal bars representing the 95 percent C I for each variable. The x-axis at the bottom is labeled lower odds to the left and higher odds for suicide ideation to the right, with a scale from negative 25 to 25.

From top to bottom, the variables and their corresponding Odds Ratio and 95 percent C I are

* Age (yrs) at 1.00 (0.90, 1.00).

* Monthly income (<25,000 I N R) at 1.20 (0.50, 2.80).

* Occupation (Begging) at 10.40 (2.50, 43.00).

* Cannabis use-Yes at 8.40 (1.60, 43.40).

* Stressful events >1 at 3.90 (1.20, 13.20).

* Gender Dysphoria at 3.70 (1.50, 9.10).

* Depression diagnosed at 3.70 (1.00, 13.20).

* Anxiety diagnosed at 12.00 (2.10, 67.20).

* Physical activity (>2hr/wk) at 3.00 (1.00, 9.30).

Variables such as Occupation (Begging), Cannabis use, and Anxiety diagnosed show the highest odds ratios and widest confidence intervals, indicating a strong but less precise association with higher odds of suicide ideation.

Navigate back to Image 1:.

